# Normal cardiac dimensions by magnetic resonance imaging and topographic anatomy of the adult arabian one-humped camel (*Camelus dromedarius)*

**DOI:** 10.1186/s12917-024-04082-z

**Published:** 2024-06-01

**Authors:** Mohamed Aref, Heba El-Zahar, Ahmed S. Mandour, Hanan H. Abd-Elhafeez, Mohsen. A Khormi, Mervat A. AbdRabou, Ahmed Abdelbaset-Ismail

**Affiliations:** 1 Department of Anatomy and Embryology, Faculty of Veterinary Medicine, Zagazig University, Zagazig, 44519 Egypt; 2 Department of Animal Medicine (Internal Medicine), Faculty of Veterinary Medicine, Zagazig University, Zagazig, Ash Sharqia, 44511 Egypt; 3 Department of Animal Medicine (Internal Medicine), Faculty of Veterinary Medicine, Suez Canal University, Ismailia, 41522 Egypt; 4 Department of Cells and Tissues, Faculty of Veterinary Medicine, Assiut University, Assiut, 71526 Egypt; 5 Department of Biology, College of Science, Jazan University, Jazan, Kingdom of Saudi Arabia; 6 Department of Biology, College of Science, Jouf University, Sakaka, Al-Jouf, Saudi Arabia; 7 Department of Surgery, Anesthesiology, and Radiology, Faculty of Veterinary Medicine, Zagazig University, Zagazig, 44519 Egypt

**Keywords:** Camelus dromedarius, MRI, Anatomy, Cardiac markers, Camel, Cardiac dimensions

## Abstract

**Background:**

Dromedaries’ normal heart architecture and size have not been adequately examined utilizing magnetic resonance imaging (MRI) and topographic anatomy.

**Result:**

we aimed to investigate the regular appearance of the heart and its dimensions, using MRI and cross-sectional anatomy, in mature Arabian one-humped camels (Camelus dromedarius). We also analyzed hematological and cardiac biochemical markers. MRI scans were conducted on twelve camel heart cadavers using a closed 1.5-Tesla magnet with fast spin echo (FSE) weighted sequences. Subsequently, the hearts were cross-sectionally sliced. Additionally, hematobiochemical studies were conducted on ten mature live camels. The study analyzed standard cardiac dimensions including HL, BW, RA, LA, RV, LV, IVS, LAD, RAD, RVD, AoD, TCVD, and MVD. The results showed a strong positive correlation between the cardiac dimensions obtained from both gross analysis and MR images, with no significant difference between them. On both gross and MRI images, the usual structures of the heart were identified and labeled. Along with the cardiac markers (creatine kinase and troponin), the average hematological values and standard biochemical parameters were also described.

**Conclusion:**

According to what we know, this investigation demonstrates, for the first time the typical heart structures and dimensions of the heart in dromedaries, and it could serve as a basis for diagnosing cardiac disorders in these animals.

## Background

Camel sports are popular in the Arab countries of the Middle East. In the future, MRI evaluation may be used to select camels and detect heart lesions.

The morphology and imaging of dromedary camel hearts is an interesting research topic with potential clinical applications. A recent study investigated the architecture of the ventricles using gross and computed tomography (CT) analysis on dromedary camel hearts. The findings of this study provide a valuable guide for future cardiac CT investigations in live camels [1].

In veterinary medicine, echocardiography and electrocardiograms are the most commonly used methods for cardiac imaging. However, magnetic resonance imaging (MRI) has emerged as a non-invasive and effective tool for cardiac imaging. It offers improved accuracy in terms of spatial and temporal image quality. This is especially important as the slice-based direction is a significant parameter for image quality [1, 2]. . MRI is a valuable tool for observing the heart’s morphology as it clarifies anatomical features clearly [3, 4]. It can help diagnose and monitor various cardiovascular diseases where other tools may not be effective [1, 5]. In the veterinary field, MRI has been extensively used to diagnose and follow-up on various cardiac diseases in small animals, particularly dogs and cats [1, 6].

Performing MRI on large animals is technically challenging due to the lack of sufficient magnetom fittings for these animals [7, 8]. This limitation has resulted in the application and interpretation of MR images of large animals, such as horses, cattle, and camels, being restricted to examining cadavers [9, 10].

As far as we know, there are no published reports that demonstrate the standard dimensions of the heart in adult dromedaries using MRI and gross anatomy. It is worth mentioning that only one article about camel calves has depicted the standard heart sizes using gross anatomy [11].

Camel racing is a popular social tradition, but camel diseases can have a major impact on both the economy and animal welfare. Diagnosing these diseases is difficult and requires accurate imaging of bones and soft tissues. While radiography and ultrasonography are commonly used, CT and MRI provide more accurate imaging of bones and soft tissues. They also allow for the evaluation of articular cartilage and small ligamentous and tendentious structures that are not easily accessible [12]. However, the limited availability and high cost of these imaging modalities make their use in camel practice challenging. All previous studies on the application of CT and MRI in dromedary camels were conducted on cadavers [13].

Accurate interpretation of a disease condition heavily depends on standard values. Therefore, determining the standard morphometric values of the heart, such as cardiac wall thickness, chamber diameter, and aortic size, is crucial in distinguishing between normal and diseased states. Several studies have reported standard cardiac dimensions in both cadavers and live individuals in humans [3, 4]. This study aimed to identify typical cardiac dimensions and describe adult camel heart structures using MRI and gross anatomy.

Meanwhile, the hematological and biochemical findings play a major role in reflecting the health status of the heart and its function, especially cardiac biomarkers such as cardiac troponin (cTn-I) which reflects the possible cardiac injury if increased [14]. Many hematological parameters such as RBCs, PCV, erythrocyte indices, and leucocytic count, and biochemical parameters including electrolytes, liver enzymes, and kidney enzymes can reflect the stressful aspects that impact the performance of camels.

## Results

### 1. Clinical examination and haemato-biochemical findings

The camels used in this study were healthy, with no history or clinical findings indicative of cardiac diseases. There were no murmurs or dysrhythmia detected by heart auscultation. The mean ± SD body temperature was 37.27 ± 1.27 ^o^C, the respiratory rate was 15.05 ± 2.59 breaths per minute, and the heart rate was 44.09 ± 8.81 beats per minute. The average hematological values obtained after analyzing adult camel blood were RBCs (11.10 ± 1.62 × 10^^6^/µL), PCV (29.45 ± 0.05%), hemoglobin (14.67 ± 0.28 g/dL), MCV (37.34 ± 1.44 femtoliters), MCH (14.69 ± 0.15 pg), MCHC (43.78 ± 0.17 g/dL), WBCs (12.20 ± 2.11 × 10^3/µL), neutrophils (53.85 ± 4.26%), lymphocytes (35.73 ± 1.46%), monocytes (5.24 ± 0.47%), eosinophils (3.5 ± 1.48%), and basophils (1.5 ± 0.48%). The average values of standard biochemical parameters were total proteins (6.4 ± 0.12 g/dL), albumin (3.37 ± 0.02 g/dL), glucose (92.16 ± 1.34 mg/dL), blood urea nitrogen (BUN; 14.84 ± 0.17 mg/dL), aspartate aminotransferase (AST; 82.14 ± 3.41 IU/L), gamma-glutamyl transferase (γGT; 18.64 ± 2.24 IU/L), creatine kinase (82.69 ± 2.02 IU/L), Cardiac troponin concentration was 0.042 ± 0.06 ng/mL, calcium (10.13 ± 0.23 mg/dL), phosphorous (5.94 ± 0.67 mg/dL), sodium (162.23 ± 0.52 mM), potassium (6.54 ± 0.07 mM), and chloride (114.23 ± 0.37 mM).

### Morphometric and morphologic findings

The heart of the camel appeared conical-shaped and was found to be located in the ventral third of the thoracic cavity between the 3rd and 5th intercostal spaces (ICS). The mean ± SD heart weight was 2.4 ± 1.4 kg. The heart weight/body weight ratio was [0.004 ± 0.0002 (kg/kg)]. The median, median range, 95% CI, and coefficient of variation of the grossly- and MRI-measured cardiac scores are summarized in Table (1).

**Table 1:** Anatomical and MRI cardiac dimensions in an adult one-humped camel

Variables	Gross measurements			MRI measurements		
	Median (median range)	95% CI of median (Lower-Upper)	CV	Median (median range)	95% CI of median (Lower-Upper)	CV
BW	150 (19)	142–156	4.4%	144.8 (24)	140–152	4.9%
RA	5.7 (1.1)	5.2-6	6.9%	5.5 (0.9)	5.1–5.7	6.1%
RV	12.6 (1.9)	12.4–13.2	5.4%	12.4 (2.1)	12.1–12.9	5.5%
LA	7.7 (1)	7.4–7.9	4.7%	7.4 (1.4)	7.1–7.6	5.9%
LV	28.6 (6.4)	26.3–30.2	7.4%	28.2 (8)	25–30	8.7%
IVS	21 (5.5)	20-24.1	8.9%	20.6 (9.8)	18.6–23	14.5%
HL	220 (13.2)	217.1-222.4	1.9%	218 (13)	215.2-220.3	1.8%
LAD	8.6 (2.5)	8-9.3	9.1%	8.3 (2.3)	7.7-9	8.7%
LVD	13.6 (6)	11–15	16.3%	13.5 (5.8)	10.7 (15.1)	16.3%
RAD	6.1 (2.5)	5.4-7	13.3%	5.8 (2.4)	5.3–6.7	13.2%
RVD	6.3 (4)	5–8	22.6%	5.8 (4)	4.9–7.8	24.2%
AoD	6.5 (3.5)	5.5–7.5	16.5%	6.1 (2.9)	5.2-7	15.4%
TCVD	4.4 (4.2)	2.5-6	34.2%	NA	NA	NA
MVD	5.7 (3)	4.8–6.8	17.4%	NA	NA	NA

Descriptive statistical analysis of the camel cardiac scores obtained from gross anatomy and MR imaging. These data represent diameter measurements (cm) measured macroscopically and on MR images. These images were collected from the cadaver hearts of clinically healthy one-humped camels (*n* = 12). There were no detectable cardiac abnormalities in the hearts of their corresponding live camels. Coefficient f variation (CV), coefficient interval (CI), Heart length (HL), heart base width (BW), and thickness of the right atrium (RA); left atrium (LA), right ventricle (RV), left ventricle (LV), and interventricular septum (IVS) were measured macroscopically and on MR images. LAD = left atrial diameter; RAD = right atrial diameter; RVD = right ventricular diameter; AoD = aortic diameter; TCVD = tricusped valve diameter; MVD = mitral valve diameter. NA = not available.

### 3. MRI findings

**Fig. 1:**
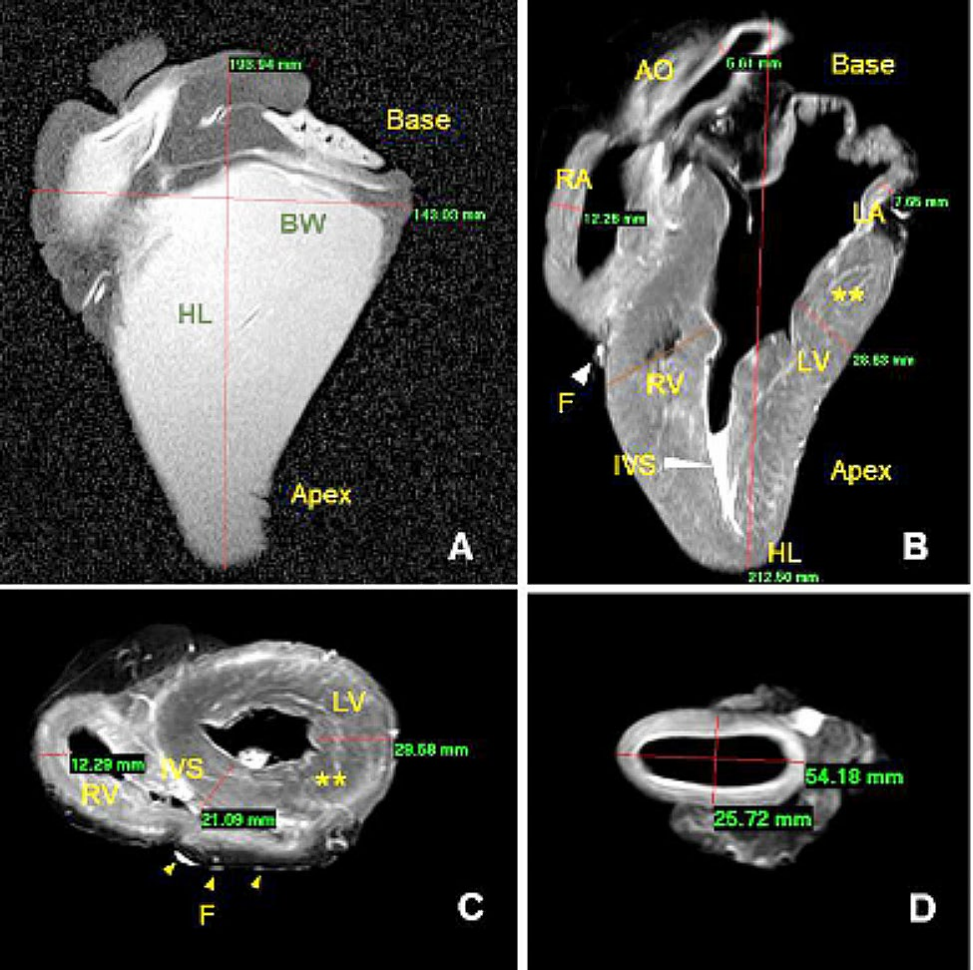
(**A**) A representative MRI image of the heart showing heart length (HL) and heart base width (BW) measurements in millimeters. (**B**) A representative axial cardiac MRI plane showing measurements of the right atrium (RA), left atrium (LA), right ventricle (RV), left ventricle (LV), and interventricular septum (IVS). High-intensity epicardial fat (**F**) and moderate-intensity myocardium (**) are also shown. (**C**) a representative transverse cardiac MRI plane showing the right ventricle (RV), left ventricle (LV), and interventricular septum (IVS). High-intensity epicardial fat (**F**) and moderate-intensity myocardium (**) are also shown. (**D**) 3D MR image showing the aortic dimensions

The normal HL, BW, RA, LA, RV, LV, IVS, LAD, RAD, RVD, and AoD were measured as shown in Table 1. The midportions of RVC, LVC, RAC, LAC, and DA were used to measure their diameters. Maximal HL was measured at the endpoints of the apex and base of the heart. Maximal CT was measured at the broadest distance between the caudal and cranial surfaces of the heart. The natural contrast of cardiac chambers and walls on MR images provided an optimal delineation for the cardiac measurements. On MR images, pericardial fat appeared bright and copious, as we observed anatomically. Compared to the transverse one, this copious fat did not allow the pericardium to be visualized in the axial plane (Fig. 1).

### 4. Anatomical findings

**Fig. 2:**
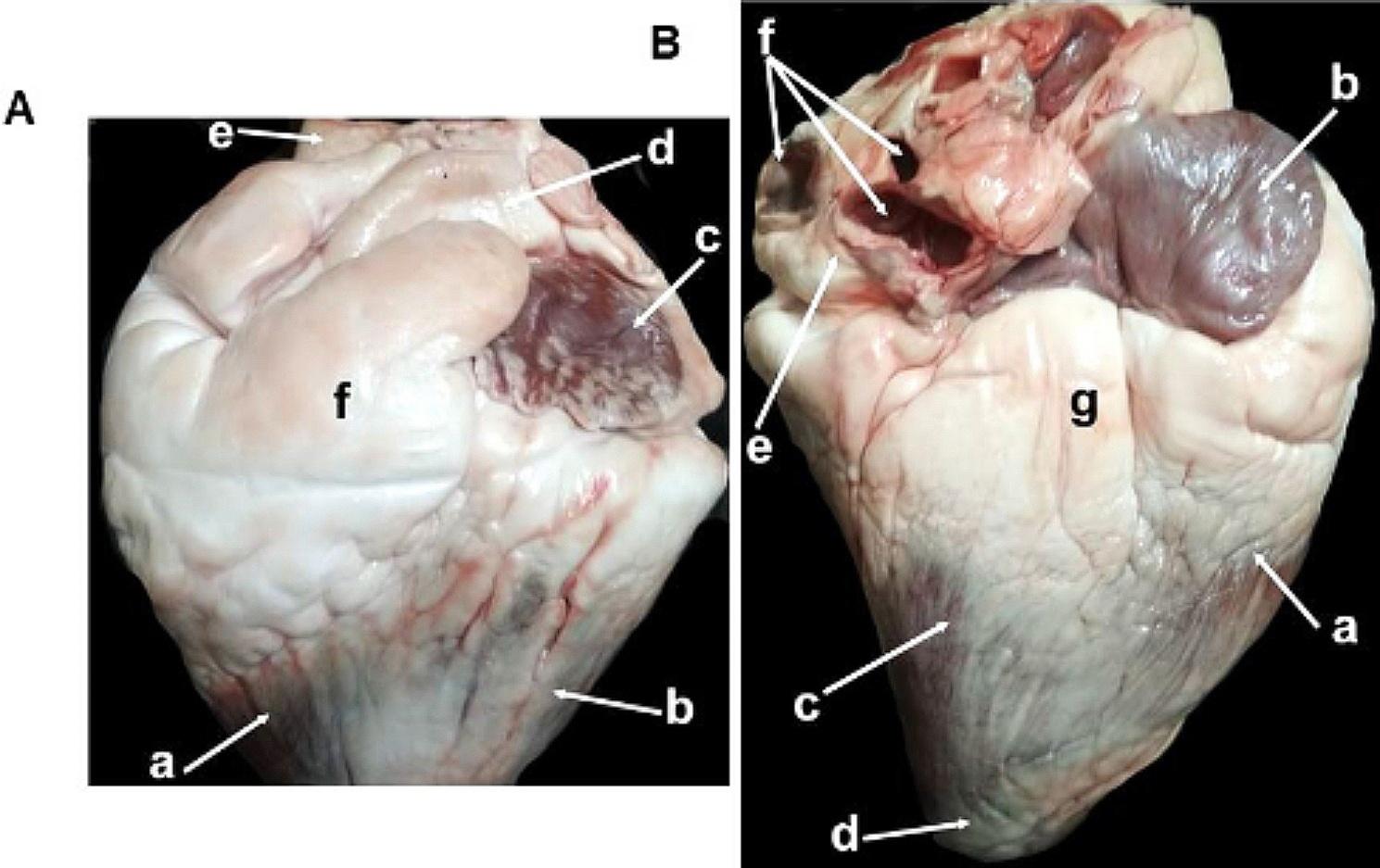
Representative macrographic images of the left (Panel **A**) and right (Panel **B**) surfaces of the heart of an adult healthy one-humped camel. Panel **A:** a-right ventricle; b-left ventricle; c-left auricle; d-pulmonary artery; e-aorta; f-coronary fat. Panel **B:** a-right ventricle; b-right auricle; c-left ventricle; d-apex; e-left auricle; f-pulmonary veins

**Fig. 3:**
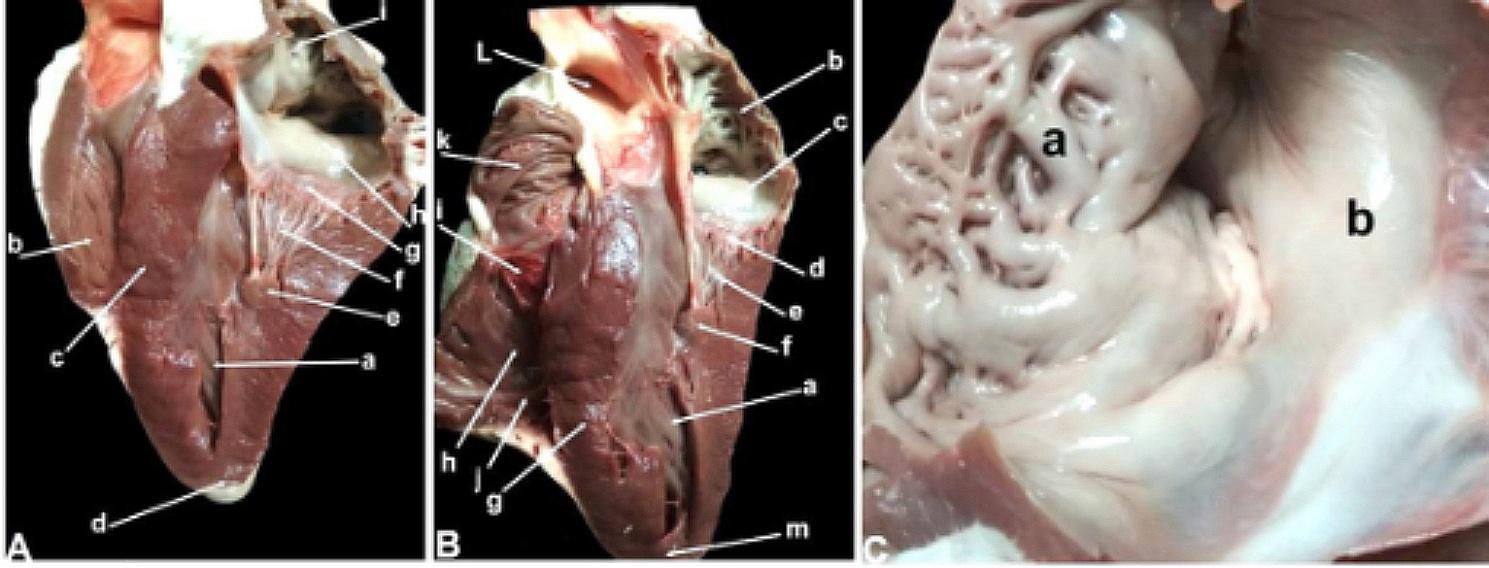
Representative macrographic images of the interior surface of the heart (Panels **A** and **B**), the interior surface of the atrium (Panel **C**), and the chordae tendineae of the right ventricle (Panel **D**) and left ventricle (Panel **E**) of an adult healthy one-humped camel. Panel **A:** a-left ventricle; b-right ventricle; c-interventricular septum; d-apex; e-papillary muscle; f-chordae tendineae; g-cusp of left atrioventricular opening; h-left atrium; i-pectinate muscle. Panel **B:** a-left ventricle; b-left auricle; c-left sinus venarum cavarum; d-cusp of left atrioventricular opening; e-chordae tendineae; f-papillary muscle; g-interventricular septum; h-right ventricle; i-cusp (flap) of right atrioventricular opening; j-moderator band; k-right auricle; l-aorta (opened). Panel **C:** a-auricle (pectinate muscle); b-sinus venarum cavarum

The cardiac structures were grossly identified and labeled, as shown in Figs. 2 and 3. The RA formed the right cranial part of the heart base and was located dorsal to the RV cranial to the origin of the pulmonary trunk. The LA formed the left caudal part of the heart base and was dorsal to the LV. Seven to eight pulmonary veins opening into the atrium caudally were found.

We also found that the left caudal portion of the ventricular size, including the cardiac apex, was formed by the LV. RV was noticed to be thinner than LV. The left and right atrioventricular (AV) orifices connected the atria and ventricles on the left and right sides, respectively. The left AV and right AV orifices had a left bicuspid (mitral) and a right tricuspid valve, respectively. The peripheral boundaries of these cusps were found attached to the AV opening. The peripheral boundaries of these cusps were found attached to the AV opening. In contrast, it appears that chordae tendineae—inelastic fibrous connective tissue strings—attach the middle edges to the ventricular wall. The chordae tendineae lines were found in the wall of the right ventricle. In the wall of the right ventricle, the chordae tendineae were found to connect the three papillary muscles to the right tricuspid valve. Two tricuspid valves were located on the interventricular septum, and the third was on the cranial ventricular wall. On the left side, the chordae tendineae appeared less in number and more prominent in size than those on the right side. Furthermore, two papillary muscles were found: one on each side. Two septo-marginal moderator bands were detected; the largest one connected the interventricular septum to papillary muscles, and the smaller one was mainly located at the apex. The trabecula carnea consisted of papillary muscle, septo-marginal band, and false tendinous. The tendinous cord, the trabecula carnea, and the fibrous rings were observed in the left and right atrioventricular orifices.

### Relationship between anatomical and MRI dimensions of a camel’s heart

**Fig. 4:**
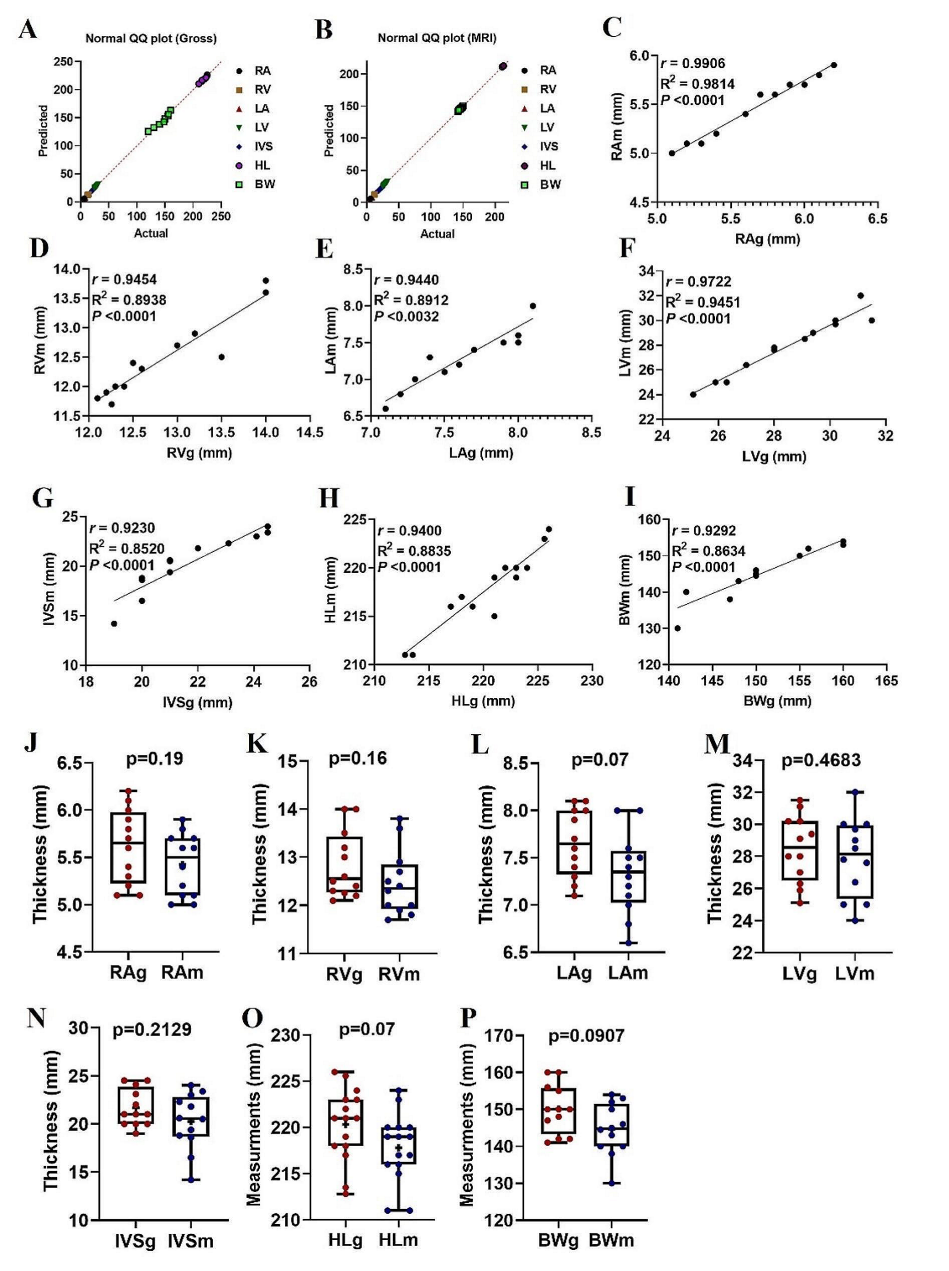
QQ plots of cardiac thickness data sets were obtained from gross (**A**) and MR images (**B**) from the normality test. Scatterplots of the thickness measurements determined from gross images versus the MRI measurements (**C-I**) obtained from 12 clinically normal dromedaries. The solid line represents the linear equation fitted to the data points; the corresponding r, R2, and *p* values are provided in each panel. Box-and-whiskers plots of RA (**J**), RV (**K**), LA (**L**), LV (**M**), IVS (**N**), HL (**O**), and BW (**P**) for hearts that had been obtained from the gross and MRI images. The line represents the median, the cross represents the mean, and the whiskers represent the range. Significant at *p* < 0.0001 and *p* < 0.05. Heart length (HL), heart base width (BW), and thickness of the right atrium (RA); left atrium (LA), right ventricle (RV), left ventricle (LV), and interventricular septum (IVS) were measured macroscopically and on MR images

**Fig. 5:**
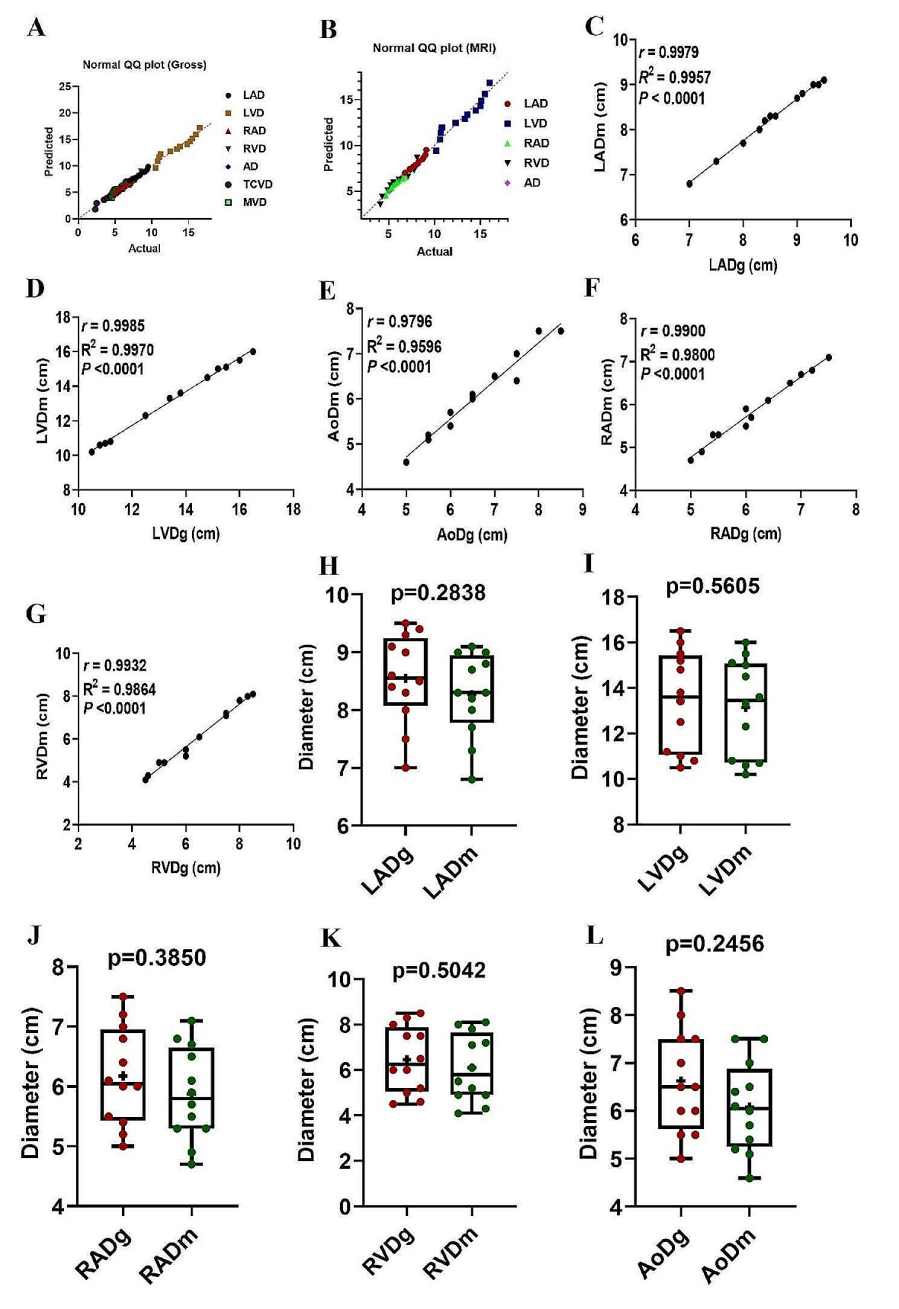
QQ plots of cardiac diameter data sets obtained from gross (**A**) and MR images (**B**) obtained from the test of normality. Scatterplots of the thickness measurements determined from gross images versus the MRI measurements (**C-G**) obtained from 12 clinically normal dromedaries. The solid line represents the linear equation fitted to the data points; the corresponding r, R2, and *p* values are provided in each panel. Box-and-whiskers plots of LAD (**H**), LVD (**I**), RAD (**J**), RVD (**K**), and AoD (**L**) for hearts that had been obtained from the gross and MRI images. The line represents the median, the cross represents the mean, and the whiskers represent the range. Significant at *p* < 0.0001 and *p* < 0.05. LAD = left atrial diameter; RAD = right atrial diameter; RVD = Right ventricular diameter; AoD = aortic diameter; TCVD = tricusped valve diameter; MVD = mitral valve diameter

We found strong correlations between the thickness (Fig. 4) and diameter (Fig. 5) data sets of the heart structures found on both gross and MR images. Also, we found no significant differences between the values measured on gross images and the corresponding values measured on MRI (Figs. 4 and 5). There were strong positive correlations found between the gross and MRI-derived thickness measurements for the RA (*r* = 0.99; *P* < 0.0001), RV (*r* = 0.95; *P* < 0.0001), LA (*r* = 0.94; *P* < 0.003), LV (*r* = 0.97; *P* < 0.0001), IVS (*r* = 0.92; *P* < 0.0001), HL (*r* = 0.94; *P* < 0.0001), and BW (*r* = 0.93; *P* < 0.0001).

At the same time, there was no significant difference in RAg versus RAm (*p* = 0.19), RVg versus RVm (*P* = 0.16), LAg versus LAm (*P* = 0.07), LVg versus LVm (*P* = 0.47), IVSg versus IVSm (*P* = 0.21), HLg versus HLm (*P* = 0.07), and BWg versus BWm (*P* = 0.09). When the diameter values were looked at, there were strong positive correlations found in RAD (*r* = 0.99; *P* < 0.0001), LVD (*r* = 0.99; *P* < 0.0001), AoD (*r* = 0.98; *P* < 0.0001), RAD (*r* = 0.99; *P* < 0.0001), or RVD (*r* = 0.99; *P* < 0.0001). At the same time, there was no significant difference in LADg versus LADm (*p* = 0.28), LVDg versus LVDm (*P* = 0.56), RADg versus RADm (*P* = 0.39), RVDg versus RVDm (*P* = 0.5), or AoDg versus AoDm (*P* = 0.25).

## Discussion

This is the first report demonstrating standard cardiac dimensions of heart cadavers on MRI and gross anatomy in adult camels. In this study, we used heart cadavers for measuring cardiac scores since the large size of the camel hinders its subjection to MR imaging owing to the absence of a sizeable fitting magneto. Consistently, several studies have been conducted using camel cadavers. The cardiac cadavers were used to assess their morphometric measurements in dromedary camel calves either grossly [11] or microscopically [15]. Of note, human cardiac cadavers were used to estimate the various cardiac dimensions [4], and other cadaveric human structures were used for MRI analysis. On the other hand, several studies examined several other camel cadaver organs, including the kidneys, adrenal glands, and vallate papillae, morphometrically [16, 17]. MRI, as beneficial diagnostic imaging, was used in the assessment of camel tarsus [18–20], normal brain [21, 22], standard head [7, 9], and normal testes and epididymis [23]. Histological, radiological, and ultrasonographic studies have also examined camel cadaveric parts [23].

A previous study has shown a mean heart weight of 1.14 ± 0.053 kg, ranging from 0.96 to 1.72 kg in camel calves [11]. In the current study, we found that the heart of adult camels weighs 2.4 ± 1.4 kg. This was consistent with that previously described in mature camels by Schwartz and Dioli (1.5–2 kg) [24].

Comparing the hematological and biochemical results obtained in this study with a previous study by [25], we confirmed that camels were healthy and had no underlying disease process affecting other results, in addition, there are no stress-related issues impacting the results of MRI and echocardiography.

Over the past decade, MRI has become well-recognized as a valuable diagnostic tool for referral in veterinary practice. It is commonly used in brain and small animal spine diseases, auditory and nasal disorders, and planning for soft tissue, neoplasm, and orthopedic surgeries for young animals and horses. MRI has grown in these disciplines due to its ability to visualize soft tissue structures [1]. Cardiac MRI has become optimal for morphological evaluation and ventricular and atrial function determination. Despite several experimental and clinical cardiac MRI scans of dogs and cats that have been performed, no veterinary investigations have yet been published in camels to compare the camel cardiac scores obtained herein. For instance, in dogs, an MRI of harvested postmortem hearts was used to identify clinical heart diseases [5], experimental heart diseases [6], and the morphology of the normal heart muscles [26]. In human clinical practice, MRI’s evaluation of cardiac morphology has become well-known as a standard reference technique. In a human cross-sectional study of cardiac cadavers, the mean values of the heart length, width, and weight were identified [4]. In another human brief report [3], the MRI dimensions of a normal heart for IVS, LVD, RVD, and descending aorta diameter were estimated. There are various disorders affecting the heart of the camel that has been diagnosed, such as pericarditis, vegetative valvular endocarditis, necrotic myocarditis, hypertrophic cardiomyopathy, and congenital defects (e.g., septal defects, patent ductus arteriosus, and sarcocystosis) [14]. The diagnosis of the aforementioned heart disease typically occurs in slaughterhouses or happens by chance during a post-mortem examination. This shows that diagnosing camel heart disease is difficult, especially when typical clinical signs of heart failure are absent.

As indicated here, the highly significant positive correlation with no significant difference between the two measurement methods suggests the reproducibility of the techniques used to evaluate camel heart dimensions. Thus, it may be necessary, particularly for further anatomical or forensic studies.

### Study limitations

The study has a few limitations that need to be considered. Firstly, a small sample size was used due to the scattering of camel populations. Secondly, we were unable to compare our findings with data from other large animals because there is a lack of literature demonstrating the heart dimensions of camels or other large animals using MRI or even gross examination. Thirdly, further research is needed to establish cardiac reference intervals by MRI within camels, taking into consideration age, sex, and body weight. Lastly, it was challenging to find cadavers of diseased camel hearts suitable for MRI and gross anatomy investigation.

## Conclusions

To the best of our knowledge, this study is the first to combine gross and magnetic resonance imaging (MRI) measurements to determine the typical heart size of dromedaries. This is due to the lack of information regarding the use of cardiac MRI to determine the typical dimensions of dromedary hearts in the veterinary literature. Anatomical sectioning and magnetic resonance imaging (MRI) in postmortem studies are thus complementary methods. Unfortunately, assessing cardiac morphology is hindered by the fact that blood flow does not demarcate the heart chambers.

## Materials and methods

### Ethical approval

This study was conducted following the rules of the ethical committee and animal welfare of the Faculty of Veterinary Medicine, Zagazig University, Egypt, and with the approval of the Institutional Animal Care and Use Committee (Approval # ZU-IACUC-2-F-163-2022) of Zagazig University. All procedures were carried out in compliance with the applicable rules and regulations. The study was conducted in accordance with the ARRIVE (Animals in Research: Reporting In Vivo Experiments) criteria [27].

### Animals and cardiac cadavers

A total of 22 clinically normal adult male Baladi one-humped camels were involved in this study. The mean age of camels was 4.2 years [standard deviation (SD): 1.1], and the mean body weight was 412.8 kg (SD: 54.1). Of these camels, 12 cadaver hearts were freshly collected from a public slaughterhouse located in El-Sharika Province, Egypt. These camels were clinically healthy without any evidence of cardiac abnormalities based on routine pre-slaughtering examinations done by registered veterinarians at the slaughterhouse and by gross examinations performed on the harvested hearts post-slaughtering. The body weight, heart rate, respiratory rate, and body temperature were recorded.

### Macroscopic examination

Immediately post-slaughter, the hearts were washed to ensure the removal of blood, and the gross measurements for cardiac length (CL), cardiac base width (BW), and thickness of the right atrium (RA), left atrium (LA), right ventricle (RV), left ventricle (LV). The interventricular septum (IVS) was measured by a vernier caliper. The CL was calculated from the apex to the base, while the BW was measured at the broadest portion between the cranial and caudal cardiac surfaces. The weights of these hearts (CW) were measured using an electronic weighing device after thoroughly cleansing heart blood. The left atrial diameter (LAD), right atrial diameter (RAD), right ventricular diameter (RVD), aortic diameter (AoD), tricuspid valve diameter (TCVD), and mitral valve diameter (MVD) were also measured.

### MR imaging

Afterward, the hearts were subjected to MR scanning within 4 h post-harvesting according to [9, 28]. The MR imaging was performed using an internal magnet of 1.5-Tesla field strength (Philips, Intra, USA). For the MR scans, fast spin echo (FSE) T1-weighted sequences, 350 ms repetition time (TR), 0.8 s echo time (ET), and one excitation (E) were used in both the transverse and axial planes. On MR images, HL, BW, RA, LA, RV, LV, IVS, LAD, RAD, RVD, and AoD were measured. A computer-based electronic light pen measured the standard cardiac dimensions of the camel on MR images, and the visualized cardiac structures were identified and labeled.

### Anatomical and morphometric examination

After completion of MR scanning, the hearts were immediately sectioned, and the anatomical morphology was identified based on the nomenclature of Nomina Anatomica Veterinaria [13]. An expert anatomist (M. A.) carried out all anatomical studies. Photo-macrographs were captured using a high-resolution digital camera (Canon).

### Biochemical and hematological studies

For biochemical and hematological investigations, 10 clinically healthy camels were also involved in this study and underwent a complete physical and clinical examination for any detectable cardiac disorders. Blood samples (10 ml volume) were collected from jugulars into EDTA-coated and non-coated microvette tubes for plasma and serum analysis. Hematological analysis for RBCs, PCV%, hemoglobin concentration, MCV, MCH, MCHC, WBCs, and the differential leucocytic count was performed within an hour of sample collection using an automated hematological analyzer (ADVIA 120, Bayer Diagnostics, Germany) according to the manufacturer’s instructions. After microcentrifugation, the obtained sera were kept on ice and stored at -20 ^o^C for subsequent biochemical analysis using a Hitachi 912 Automatic Analyzer (Roche Diagnostics GmbH, Mannheim) according to the manufacturer’s instructions. The following parameters were measured: glucose, total protein, albumin (ALB), blood urea nitrogen (BUN), creatine kinase (CK), aspartate aminotransferase (AST), α glutamyl transferase (αGT), calcium (Ca), phosphorous, sodium (Na), potassium (K), and chloride (Cl). In addition, the concentration of serum cardiac troponin I (cTn-I) was determined with a commercial kit (Card-Ikit Combo Test; Aboa Tech).

### Statistical analysis

Before starting the analyses, the data set in each variable was tested for normal (Gaussian) distribution with the D’Agostino-Pearson omnibus normality test, and standard QQ plots were created. Descriptive analysis was also performed for all data sets, and the obtained data were displayed as mean ± SD and with 95% confidence intervals (CIs) of the mean. An unpaired 2-tailed t-test was employed to compare the means of cardiac scores obtained by gross measurements versus measurements obtained by MRI. The r values were computed between selected data sets. A Pearson correlation coefficient (r) and R2 values were calculated to evaluate the relationship between cardiac scores measured on gross examination and the corresponding dataset derived from MRI. Data was analyzed using Prism GraphPad Software-version 7 (GraphPad Software Inc, California). For all analyses, statistical significance was set at *p* < 0.05.

## Data Availability

All data obtained is included in this manuscript and is available on request from the corresponding authors. There is no sequence data in this manuscript.
